# Bundle Block Adjustment of Airborne Three-Line Array Imagery Based on Rotation Angles

**DOI:** 10.3390/s140508189

**Published:** 2014-05-07

**Authors:** Yongjun Zhang, Maoteng Zheng, Xu Huang, Jinxin Xiong

**Affiliations:** School of Remote Sensing and Information Engineering, Wuhan University, Wuhan 430079, China; E-Mails: tengve@whu.edu.cn (M.Z.); huangxu.chess@163.com (X.H.); xalson2014@gmail.com (J.X.)

**Keywords:** airborne three-line array imagery, block adjustment, rotation angle, systematic error compensation, orientation image

## Abstract

In the midst of the rapid developments in electronic instruments and remote sensing technologies, airborne three-line array sensors and their applications are being widely promoted and plentiful research related to data processing and high precision geo-referencing technologies is under way. The exterior orientation parameters (EOPs), which are measured by the integrated positioning and orientation system (POS) of airborne three-line sensors, however, have inevitable systematic errors, so the level of precision of direct geo-referencing is not sufficiently accurate for surveying and mapping applications. Consequently, a few ground control points are necessary to refine the exterior orientation parameters, and this paper will discuss bundle block adjustment models based on the systematic error compensation and the orientation image, considering the principle of an image sensor and the characteristics of the integrated POS. Unlike the models available in the literature, which mainly use a quaternion to represent the rotation matrix of exterior orientation, three rotation angles are directly used in order to effectively model and eliminate the systematic errors of the POS observations. Very good experimental results have been achieved with several real datasets that verify the correctness and effectiveness of the proposed adjustment models.

## 1. Introduction

The principle of three-line scanner imagery was first proposed by Hoffman *et al.* [1]. The problem of the low vertical accuracy of adjustment, caused by the traditional small format images, was significantly improved by this method and has been successfully applied in the German MOMS02 project with very good results [2–4]. In recent years, all of the high resolution Earth observation satellites, such as SPOT5 [5,6], QuickBird [7,8], IKONOS [7–9], and more recently WorldView and GeoEye *etc.*, have utilized a linear push broom scanner to collect image data. Three-line scanners have been widely exploited not only in satellite photogrammetry, but also in aerial photogrammetry. In 2000, LH Systems successfully applied the three-line imagery technology in aerial photogrammetry and announced the airborne digital three-line scanner ADS40 [7,10,11]; and several airborne digital three-line scanners were released thereafter, such as StarImager [12–14] and 3-DAS-1 [14]. These airborne digital three-line scanners usually have a global positioning system (GPS) and inertial measurement unit (IMU) on board that can acquire the exterior orientation parameters (EOPs) with considerable precision and provide more observations for bundle adjustment. Under these conditions, the coordinates of ground objects can be precisely determined with only four control points distributed at the four corners, thereby providing a new solution for the automation of photogrammetry. The related processes such as image data preprocessing, triangulation [15–17] and fast generation of digital surface model (DSM) [6,18,19] have been thoroughly investigated. GPS/IMU aided processing of airborne three-line imagery becomes one of the central issues in aerial photogrammetry.

Image data acquired by three-line scanners have a large base-height ratio, but the linear array push broom strategy makes the EOPs of scanning lines vary by time, and each scanning line thus has different EOPs. It is impossible to simultaneously solve the EOPs of all of the lines in bundle adjustment [1]. In order to reduce the unknowns, a proper sensor model must be established to represent the position and attitude of the sensor. Several sensor models, for example, the direct geo-referencing model [20–22], the piecewise polynomial model [1,23] and the orientation image model [1,13,24], have been proposed by researchers. The introduction of IMU has provided a great number of useful observations. Many researches to date have focused on using the *φ-ω-κ* rotation angle system defined by sequential rotations about the Y, X, and Z axes or a quaternion [15–17,24] to represent the attitude parameters. However, the attitude data output from IMU are usually based on the *ω-φ-κ* rotation angle system (or namely the roll, pitch and yaw system) defined by sequential rotations about the X, Y, and Z axes [25]. For better modeling the systematic error of IMU, this paper employs the *ω-φ-κ* rotation angle system and adopts both the systematic error compensation model and the orientation image model in the bundle adjustment. Four real datasets are used for our experiments, and the accuracies of both the systematic error compensation model and the orientation image model are discussed. The achieved results are also compared with the available results in the literature based on *ω-φ-κ* rotation angle system and quaternion.

## 2. Mathematical Model of Bundle Block Adjustment

### 2.1. Basic Theory

As previously mentioned, it is impossible to set the EOPs of all of the scanning lines as unknowns during bundle adjustment. Proper sensor models are necessary to approximate these unknown parameters. Generally, the direct geo-referencing model, systematic error compensation model, piecewise polynomial model, and orientation image model are often adopted. The standard output of IMU is based on the *ω-φ-κ* rotation angle system (or roll, pitch and yaw system) [24]. Therefore, only in the case where the recommended *ω-φ-κ* system is adopted in bundle adjustment can the identified systematic error in the IMU observations be eliminated via systematic error modeling. Detailed proof will be given in the next section.

Regardless of which sensor model is chosen, the object point, the corresponding image point, and the photographic center should lie on the same line. Given the EOPs (*X_Sj_*, *Y_Sj_*, *Z_Sj_*, *ω_j_*, *φ_j_*, *κ_j_*) of the *j* th scanning line, the instantaneous collinearity equation of the push broom imaging system can be expressed as follows:

(1) $$\begin{array}{c} x-x_{0}=-f\frac{a_{1}\left(X-Xs_{j}\right)+b_{1}\left(Y-Ys_{j}\right)+c_{1}\left(Z-Zs_{j}\right)}{a_{3}\left(X-Xs_{j}\right)+b_{3}\left(Y-Ys_{j}\right)+c_{3}\left(Z-Zs_{j}\right)}\\ y-y_{0}=-f\frac{a_{2}\left(X-Xs_{j}\right)+b_{2}\left(Y-Ys_{j}\right)+c_{2}\left(Z-Zs_{j}\right)}{a_{3}\left(X-Xs_{j}\right)+b_{3}\left(Y-Ys_{j}\right)+c_{3}\left(Z-Zs_{j}\right)} \end{array}$$

where (*x*, *y*) are the image plane coordinates of the image point; (*x*_0_, *y*_0_, *f*) are the interior elements; (*X*, *Y*, *Z*) are coordinates of the object point; (*X_Sj_*, *Y_Sj_*, *Z_Sj_*) are the translation parameters of the scanning line; and *a_i_*, *b_i_*, *c_i_* (*i* = 1, 2, 3) are the nine elements of the rotation matrix computed by the three angles (*ω_j_*, *φ_j_*, *κ_j_*) of the scanning line.

### 2.2. Triangulation Based on the Systematic Error Compensation Model

The positioning and orientation system (POS) integrated on the three-line digital scanner often has good precision and high stability. The post-processing modules will compensate for the offset related to the projection center and the misalignment related to the IMU main axes, but many experiments have shown that some residual systematic errors will remain in the corrected POS data [23]. It can be assumed to mainly consist of GPS and IMU systematic drift errors that vary by time. Taking the GPS antenna offset errors and the IMU primary axes misalignment errors into consideration, strip related constant and linear terms are used in this paper to represent the offset and drift errors of the GPS/IMU observations. The mathematical model to calculate the photographic center (*X_S_*, *Y_S_*, *Z_S_*) with GPS observations (*X_GPS_*, *Y_GPS_*, *Z_GPS_*) is described as follows [26]:

(2) $$\left(\begin{array}{c} X_{S}\\ Y_{S}\\ Z_{S} \end{array}\right)=\left(\begin{array}{c} X_{\text{GPS}}\\ Y_{\text{GPS}}\\ Z_{\text{GPS}} \end{array}\right)-\text{R}\left(\begin{array}{c} u\\ v\\ w \end{array}\right)-\left(\begin{array}{c} a_{X}\\ a_{Y}\\ a_{Z} \end{array}\right)-\left(t-t_{0}\right)\left(\begin{array}{c} b_{X}\\ b_{Y}\\ b_{Z} \end{array}\right)$$

where (*u*, *v*, *w*) are the remaining errors of the GPS antenna offset components; **R** is the rotation matrix of the angular elements (*ω*, *φ*, *κ*); (*a_X_*, *a_Y_*, *a_Z_*) and (*b_X_*, *b_Y_*, *b_Z_*) are the systematic offset and drift errors of GPS observations of each strip; *t* is the imaging time of a certain scanning line; and *t*_0_ is the reference time that usually set to be the center time of a strip.

Calculation of the angular elements involves the rotation matrix **R***_IMU_* derived from IMU observations (*ω_I_*, *φ_I_*, *κ_I_*), the misalignment matrix **R***_MIS_* derived from the remaining misalignment errors (*ω_M_*, *φ_M_*, *κ_M_*) of IMU primary axes, and the rotation matrix **R** derived from angular elements (*ω*, *φ*, *κ*) of the EOPs. These three rotation matrices are all based on the *ω-φ-κ* rotation angle system and have the following relationship [24]:

(3) $$\text{R}={\text{R}}_{\text{MIS}}^{\text{T}}{\text{R}}_{\text{IMU}}$$

Considering the strip offset parameters (*a_ω_*, *a_φ_*, *a_κ_*), and first order drift parameters (*b_ω_*, *b_φ_*, *b_κ_*) of the IMU observations, the systematic error compensated IMU observations ( $\omega_{I}^{\prime }$, $\varphi_{I}^{\prime }$, $\kappa_{I}^{\prime }$,) used in this paper is modeled as follows [26]:

(4) $$\left(\begin{array}{c} \omega_{I}^{\prime }\\ \varphi_{I}^{\prime }\\ \kappa_{I}^{\prime } \end{array}\right)=\left(\begin{array}{c} \omega_{I}\\ \varphi_{I}\\ \kappa_{I} \end{array}\right)+\left(\begin{array}{c} a_{\omega }\\ a_{\varphi }\\ a_{\kappa } \end{array}\right)+\left(t-t_{0}\right)\left(\begin{array}{c} b_{\omega }\\ b_{\varphi }\\ b_{\kappa } \end{array}\right)$$

where *t* is the imaging time of a certain scanning line; and *t*_0_ is the reference time of a strip.

The unknowns of the bundle adjustment include the remaining errors of the GPS antenna offset, the remaining misalignment errors of IMU primary axes, the strip offset, and the drift errors of the GPS and IMU observations, and the space coordinates of the object points. Given an area with m strips and n object points, the amount of unknowns is 3 + 3 + 12 × m + 3 × n. After adjustment with the least squares principle, the exact values of the systematic errors can be obtained to refine the EOPs of all of the scanning lines.

The proof of using the *ω-φ-κ* rotation angle system to model the systematic errors of IMU observations will be given by a numerical example. Suppose the IMU observations (*ω_I_*, *φ_I_*, *κ_I_*), systematic errors (*a_ω_*, *a_φ_*, *a_κ_*), and (*b_ω_*, *b_φ_*, *b_κ_*) are *ω_I_* = 0.050000, *ω_I_* = 0.050000, *κ_I_* = 1.550000; *a_ω_* = 1.0*e*^−3^, *a_φ_* = 1.0*e*^−3^, *a_κ_* = 1.0*e*^−3^; and *b_ω_* = 5.0*e*^−6^, *b_φ_* = 5.0*e*^−6^, *b_κ_* = 5.0*e*^−6^. According to the characteristics of IMU, the flying time of each strip is usually no longer than 20 min, so the maximum time offset of a scanner line is *t* − *t*_0_ = 600.00 seconds since *t*_0_ is usually set to be the center line time of a strip.

According to the above supposed values, the systematic error compensated three angles ( $\omega_{I}^{\prime }$, $\varphi_{I}^{\prime }$, $\kappa_{I}^{\prime }$,) can be calculated as follows:

(5) $$\left(\begin{array}{c} \omega_{I}^{\prime }\\ \varphi_{I}^{\prime }\\ \kappa_{I}^{\prime } \end{array}\right)=\left(\begin{array}{c} 0.050000\\ 0.050000\\ 1.550000 \end{array}\right)+\left(\begin{array}{c} 1.0e^{-3}\\ 1.0e^{-3}\\ 1.0e^{-3} \end{array}\right)+600.00\left(\begin{array}{c} 5.0e^{-6}\\ 5.0e^{-6}\\ 5.0e^{-6} \end{array}\right)=\left(\begin{array}{c} 0.054000\\ 0.054000\\ 1.554000 \end{array}\right)$$

**R***_IMU_* can be derived from the IMU observations (*ω_I_*, *φ_I_*, *κ_I_*), and ${\text{R}}_{\text{IMU}}^{\prime }$ can be computed by the systematic error compensated three angles ( $\omega_{I}^{\prime }$, $\varphi_{I}^{\prime }$, $\kappa_{I}^{\prime }$,).

(6) $$\begin{array}{l} {\text{R}}_{\text{IMU}}=\left(\begin{array}{ccc} 0.0207688 & ‐0.9985343 & ‐0.0499792\\ 0.9984824 & 0.0232662 & ‐0.0499167\\ 0.0510064 & ‐0.0488666 & 0.9975021 \end{array}\right),\\ {\text{R}}_{\text{IMU}}^{\prime }=\left(\begin{array}{ccc} 0.0167710 & ‐0.9984015 & ‐0.0539738\\ 0.9983526 & 0.0196838 & ‐0.0538951\\ 0.0548713 & ‐0.0529810 & 0.9970868 \end{array}\right) \end{array}$$

Two sets of three angles (*φ_T_*, *ω_T_*, *κ_T_*), and ( $\varphi_{T}^{\prime }$, $\omega_{T}^{\prime }$, $\kappa_{T}^{\prime }$) under the definition of the *φ-ω-κ* rotation angle system can be decomposed from the above computed rotation matrix **R***_IMU_* and ${\text{R}}_{\text{IMU}}^{\prime }$, respectively:

(7) $$\begin{array}{ccc} \varphi_{T}=0.050062, & \omega_{T}=0.049937, & \kappa_{T}=1.547499\\ \varphi_{T}^{\prime }=0.054079, & \omega_{T}^{\prime }=0.053921, & \kappa_{T}^{\prime }=1.551082 \end{array}$$

If the computed systematic errors are directly used as those under the definition of the *φ-ω-κ* rotation angle system, we can easily deduce the following three angles ( $\varphi_{C}^{\prime }$ $\omega_{C}^{\prime }$, $\kappa_{C}^{\prime }$):

(8) $$\left(\begin{array}{c} \varphi_{C}^{\prime }\\ \omega_{C}^{\prime }\\ \kappa_{C}^{\prime } \end{array}\right)=\left(\begin{array}{c} \varphi_{T}\\ \omega_{T}\\ \kappa_{T} \end{array}\right)+\left(\begin{array}{c} a_{\varphi }\\ a_{\omega }\\ a_{\kappa } \end{array}\right)+\left(t-t_{0}\right)\left(\begin{array}{c} b_{\varphi }\\ b_{\omega }\\ b_{\kappa } \end{array}\right)=\left(\begin{array}{c} 0.054062\\ 0.053937\\ 1.551499 \end{array}\right)$$

Thus we can get the differences among the two sets of three angles: $\varphi_{T}^{\prime }-\varphi_{C}^{\prime }=0.000017$, $\omega_{T}^{\prime }-\omega_{C}^{\prime }=0.000016$, $\kappa_{T}^{\prime }-\kappa_{C}^{\prime }=0.000417$. The focus to pixel size ratio of ADS40/80 sensors is always about 62.7/0.0065 = 9,630, so the maximum influence of pitch and roll errors caused by using different rotation angles is 0.000017 × 9,630 = 0.16 pixels, which usually can be neglected. However, the maximum influence of the yaw error at the image border is 6,000 × 0.000417 = 2.50 pixels considering the CCD length of ADS 40/80 sensor is 12,000 pixels, which is obviously unable to be neglected. This comparison shows that using the *φ-ω-κ* rotation angle system will cause errors of about 2.50 pixels at the image border, because the strip-related constant and drift errors in IMU observations cannot be correctly treated under this angular system. Up to now, it is clear that modeling the systematic errors of IMU observations based on the *ω-φ-κ* rotation angle system is advantageous since the standard output of IMU device is under this angular system.

### 2.3. Triangulation Based on the Orientation Image Model

The basic principle of the orientation image model is to solve the three translation and three rotation elements of the EOPs at each orientation image by bundle adjustment. The EOPs of any scanning line between the two EOPs of the orientation images can be interpolated. First or third order Lagrange interpolation [24] is commonly used to interpolate the EOPs for airborne three-line imagery. The POS observations of an airborne three-line scanner have small random errors within a short time. Therefore, these observations can be used in the interpolation of EOPs.

Given a certain ground point Pi, the corresponding image point (*x_ij_*, *y_ij_*) and the EOPs (*X_Sj_*, *Y_Sj_*, *Z_Sj_*, *ω_j_*, *φ_j_*, *κ_j_*) of the corresponding scanning line *j*, the collinearity relationship among them can be described with the EOPs of two adjacent orientation images k and *k* + 1 as follows [24]:

(9) $$\begin{array}{c} x_{ij}={\text{F}}_{ij}(X_{i},Y_{i},Z_{i},\text{Xsk},\text{Ysk},\text{Zsk},\omega_{k},\varphi_{k},\kappa_{k},\text{Xsk}+1,\text{Ysk}+1,\text{Zsk}+1,\omega_{k+1},\varphi_{k+1},\kappa_{k+1})\\ y_{ij}={\text{G}}_{ij}(X_{i},Y_{i},Z_{i},\text{Xsk},\text{Ysk},\text{Zsk},\omega_{k},\varphi_{k},\kappa_{k},\text{Xsk}+1,\text{Ysk}+1,\text{Zsk}+1,\omega_{k+1},\varphi_{k+1},\kappa_{k+1}) \end{array}$$

where **F***_ij_* and **G***_ij_* represent the collinearity equation of image coordinates (*x_ij_*, *y_ij_*), (*X_i_*, *Y_i_*, *Z_i_*) are the spatial coordinates of point Pi, (*X_Sk_*, *Y_Sk_*, *Z_Sk_*, *ω_k_*, *φ_k_*, *κ_k_*) and (*X_Sk_*_+1_, *Y_Sk_*_+1_, *Z_Sk_*_+1_, *ω_k_*_+1_, *φ_k_*_+1_, *κ_k_*_+1_) are the EOPs of two adjacent orientation images *k* and *k* + 1.

During the adjustment process, (*X_Sj_*, *Y_Sj_*, *Z_Sj_*, *ω_j_*, *φ_j_*, *κ_j_*) can be calculated by the EOPs of two adjacent orientation images and the correction values which can be derived by GPS/IMU observations. A variable *C_j_* is introduced to represent the relationship among the EOPs at time *t_j_* and the EOPs of the adjacent orientation images at time *t_k_* and *t_k_*_+1_:

(10) $$C_{j}=\frac{t_{k+1}-t_{j}}{t_{k+1}-t_{k}}$$

**Figure 1.**
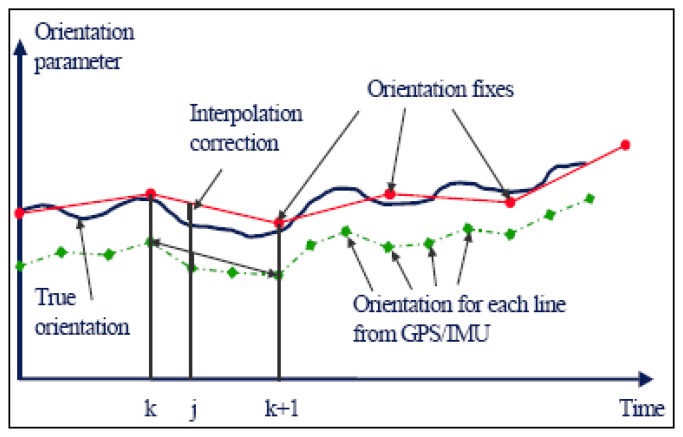
Example of one orientation parameter over time [24].

Thus (*X_Sj_*, *Y_Sj_*, *Z_Sj_*, *ω_j_*, *φ_j_*, *κ_j_*) can be calculated by the following equations [24]:

(11) $$\begin{array}{c} X_{S_{j}}=C_{j}X_{S_{k}}+(1-C_{j})X_{S_{k+1}}-\Delta X_{S_{j}}\\ Y_{S_{j}}=C_{j}Y_{S_{k}}+(1-C_{j})Y_{S_{k+1}}-\Delta Y_{S_{j}}\\ Z_{S_{j}}=C_{j}Z_{S_{k}}+(1-C_{j})Z_{S_{k+1}}-\Delta Z_{S_{j}}\\ \omega_{j}=C_{j}\omega_{k}+(1-C_{j})\omega_{k+1}-\Delta \omega_{j}\\ \varphi_{j}=C_{j}\varphi_{k}+(1-C_{j})\varphi_{k+1}-\Delta \varphi_{j}\\ \kappa_{j}=C_{j}\kappa_{k}+(1-C_{j})\kappa_{k+1}-\Delta \kappa_{j} \end{array}$$

where the correction vector Δ*X_j_*, Δ*Y_j_*, Δ*Z_j_*, Δ*ω_j_*, Δ*φ_j_*, Δ*κ_j_*, of the EOPs can be calculated by the EOPs at time *j*, *k*, *k* + 1 directly obtained from the GPS/IMU observations, as shown in Figure 1 [24]:

(12) $$\begin{array}{c} \Delta X_{j}=C_{j}X_{k}^{\text{GPS}}+(1-C_{j})X_{k+1}^{\text{GPS}}-X_{j}^{\text{GPS}}\\ \Delta Y_{j}=C_{j}Y_{k}^{\text{GPS}}+(1-C_{j})Y_{k+1}^{\text{GPS}}-Y_{j}^{\text{GPS}}\\ \Delta Z_{j}=C_{j}Z_{k}^{\text{GPS}}+(1-C_{j})Z_{k+1}^{\text{GPS}}-Z_{j}^{\text{GPS}}\\ \Delta \omega_{j}=C_{j}\omega_{k}^{\text{IMU}}+(1-C_{j})\omega_{k+1}^{\text{IMU}}-\omega_{j}^{\text{IMU}}\\ \Delta \varphi_{j}=C_{j}\varphi_{k}^{\text{IMU}}+(1-C_{j})\varphi_{k+1}^{\text{IMU}}-\varphi_{j}^{\text{IMU}}\\ \Delta \kappa_{j}=C_{j}\kappa_{k}^{\text{IMU}}+(1-C_{j})\kappa_{k+1}^{\text{IMU}}-\kappa_{j}^{\text{IMU}} \end{array}$$

The POS observations can be easily integrated into bundle adjustment with the orientation image model. The mathematical model is quite similar to the model described in the above section except that only the offset and drift parameters of the GPS/IMU observations should be taken into consideration. The unknowns of bundle adjustment now include the EOPs of the orientation images, the remaining GPS antenna bias components, the remaining IMU misalignment components, the offset and drift parameters of the GPS and IMU observations, and the coordinates of the ground objects.

The existing strategies for bundle adjustment of three-line imagery typically adopt a quaternion [15,24] or Lagrange interpolation [13,18] to represent the attitude parameters. There is a constraint condition among the four elements of a quaternion. Usually, there are two solutions to deal with the constraint. The first solution is keeping the four unknowns and one additional constraint during bundle adjustment, and the second solution is computing the fourth element with the other three elements calculated by bundle adjustment.

In this paper, bundle adjustment is realized based on the *ω-φ-κ* rotation angle system. The derivation of unknown coefficients related to translation elements is the same as that of the conventional method, whereas the derivation of unknown coefficients of IMU misalignment components (*ω_M_*, *φ_M_*, *κ_M_*) and attitude parameters (*ω_k_*, *φ_k_*, *κ_k_*), (*ω_k_*_+1_, *φ_k_*_+1_, *κ_k_*_+1_) should be obtained by linearizing the collinearity equations according to the *ω-φ-κ* rotation angle system.

Given an block area with m strips, n unknown ground points and 1 orientation images, the amount of unknowns of bundle adjustment is 3 + 3 + 12 × m + 3 × n + 6 × 1. The number of unknowns is extremely large during bundle adjustment, and thus the compression of normal equation and a fast matrix inversion algorithm are needed. EOPs of each scanning line can be interpolated by the corrected EOPs of orientation images.

### 2.4. Resolving the Interpolation Problem of Rotation Angles

The range of angles in the three-line sensor data is usually between (−*π*, *π*), therefore, the periodic issues of angle interpolation must be solved when rotation angle is used to represent the attitude of the sensor. The values of roll *ω* and pitch *φ* are definitely between (−*π*/4, *π*/4) in all cases since the optical axis of the image sensor is always pointing to the ground, but the yaw angle *k* is closely related to the flight direction of the plane. For instance, when the plane flies in the direction of south to north or north to south, *k* is usually around ±*π*. The unconformity of the sign of yaw angles usually leads to wrong interpolated values. Actually, it is impossible for the plane to make a sharp turn that causes a big variation of *k* up to 180 degrees during a small period of time (the time interval between two orientation images). Hence, a simple but effective strategy by making the two angles have the same sign is proposed to resolve the interpolation problem. Given $k_{k}^{\text{IMU}}$, $k_{k+1}^{\text{IMU}}$ as the yaw angles of two orientation images at time *k*, and *k* + 1, the yaw angle $k_{t_{j}}^{\text{IMU}}$ at time *t_j_* can be correctly interpolated by preprocessing of $k_{k+1}^{\text{IMU}}$ according to the following criteria:

(13) $$\{\begin{array}{ccccc} k_{k+1}^{\text{IMU}}=k_{k+1}^{\text{IMU}}-2\pi , & if & k_{k}^{\text{IMU}}\leq -\pi /2 & \text{and} & k_{k+1}^{\text{IMU}}\geq \pi /2\\ k_{k+1}^{\text{IMU}}=k_{k+1}^{\text{IMU}}+2\pi , & f & k_{k}^{\text{IMU}}\geq \pi /2 & \text{and} & k_{k+1}^{\text{IMU}}\leq -\pi /2 \end{array}$$

The proposed mathematical model is simpler than that uses quaternion since the constraint condition among the four elements of a quaternion can be removed, which makes dealing with large dataset possible, although the computational complexity is increased. Moreover, this strategy is advantageous for the improvement of current bundle adjustment software that adopts rotation angles.

## 3. Experiments and Analysis

A bundle adjustment software program of airborne three-line scanner imagery named iBundle-AeroTLS^®^ based on the proposed models was developed. In order to verify the correctness and effectiveness of the two models, ADS40 images collected from the Taigu and Pingyao test areas in China and the Waldkirch test area in Switzerland were used for experiments. The focal length and physical pixel size of ADS40 camera are 62.7 mm and 0.0065 mm, respectively. The adjustment results of the three test areas have been reported in papers [16,17] and are used for comparison in this paper. According to the characteristics of the ADS40 sensor [24], the time interval of the orientation image model was set at eight seconds during bundle adjustment. Additionally, the literature indicates that only four control points at four corners of the block are needed to obtain satisfactory results. Thus, the four control point strategy was adopted for all three experiments.

The ADS40 images of the Taigu test area were captured in May 2007. The entire test area consists of 13 strips and covers an area of about 30 km^2^. The ground sampling distance (GSD) is about 0.06 m while the flying height is about 600 m. A total of 105 ground control points (GCPs) were measured by differential GPS. Four control points on the four corners were used in the bundle adjustment and the other 101 points were used as check points. A total of 96,330 pass points were automatically matched by the photogrammetric software DPGrid developed by Wuhan University [27]. The bundle adjustment statistics for the two models are shown in Table 1, where RMSE is the root mean squared error of control and check point residuals, Mean and Max are the average and maximum of the residuals. The standard errors of unit weight in image space are 0.0042 mm and 0.0035 mm, respectively.

The ADS40 images of the Pingyao test area were captured in October 2006. The entire test area consists of nine strips and covers an area of about 240 km^2^. The GSD is about 0.2 m while the flying height is about 2000 m. A total of 84 GCPs were measured by differential GPS. Four control points were also used in the bundle adjustment, and the other 80 points are used as check points. A total of 54,792 pass points were automatically matched by DPGrid. The bundle adjustment statistics for the two models are shown in Table 2. The standard errors of unit weight in image space are 0.0035 mm and 0.0030 mm, respectively.

The ADS40 images of the Waldkirch test area with four east-west strips and two north-south strips were captured in May 2002. The ground coverage of this test area is about 80 km^2^. The GSD of the imagery is 0.2 m while the flying height is about 2000 m and the number of measured GCPs is 30, respectively. All of the automatically matched 25,140 conjugate points among the six strips were used in the adjustment. Four control points at the four corners were used to perform the bundle adjustment, and the other 26 points were used as check points. The bundle adjustment statistics for the two models are shown in Table 3. The standard errors of unit weight in image space are 0.0038 mm and 0.0035 mm, respectively.

In the above three experiments, the differences of the estimated misalignments of GPS and IMU obtained by the two approaches are well below 0.02 m. However, the position and attitude elements of EOPs of the orientation image model have changed a few centimeters and several arc seconds respectively when compared with those of the systematic error compensation model. As shown in Tables 1, 2 and 3, the RMSE values of the check points in planimetry were smaller than 0.7 GSD, and the maximum residuals ranged from 0.7 GSD to 2.0 GSD after bundle adjustment with the systematic error compensation model in the three test areas. The RMSE values in the height direction were slightly better than 1.0 GSD, and the maximum residuals were about 1.3∼2.5 GSD. However, some residual systematic errors remained in both the planimetry and height. After bundle adjustment with the orientation image model, the systematic components of the check point residuals were significantly reduced, for example the absolute mean value of height residuals of check points in Table 3 decreases from 0.070 m to 0.021 m. The RMSEs in the height direction substantially decreased to smaller than 0.75 GSD in the three test areas. Moreover, the maximum residuals in both the horizontal and vertical directions were smaller than 2.0 GSD, and the remained residuals show random distribution.

The results of combining both the systematic error compensation model and orientation image model were also listed in the three tables. It was shown that about 5 to 10 percent improvements were achieved when compared with using the orientation image model only.

The available results of bundle adjustment for the three test areas in the literature [16,17] were listed in Table 4. By comparison among the results listed in the four tables, we can see that the accuracies of bundle adjustment with the systematic error compensation model for the three test areas were equivalent to those with the commercial software ORIMA. However, the accuracies of bundle adjustment with the orientation image model were better than those calculated by ORIMA. For example, the RMSEs of height residuals were 0.046 m and 0.148 m in Tables 1–2 respectively, while they were 0.06 m and 0.19 m in Table 4, which means that the results of our approach with the orientation image model are up to 20 percent higher than those of the literature. These satisfying results verify the correctness and feasibility of the proposed two adjustment models based on *ω-φ-κ* rotation angle system.

**Figure 2.**
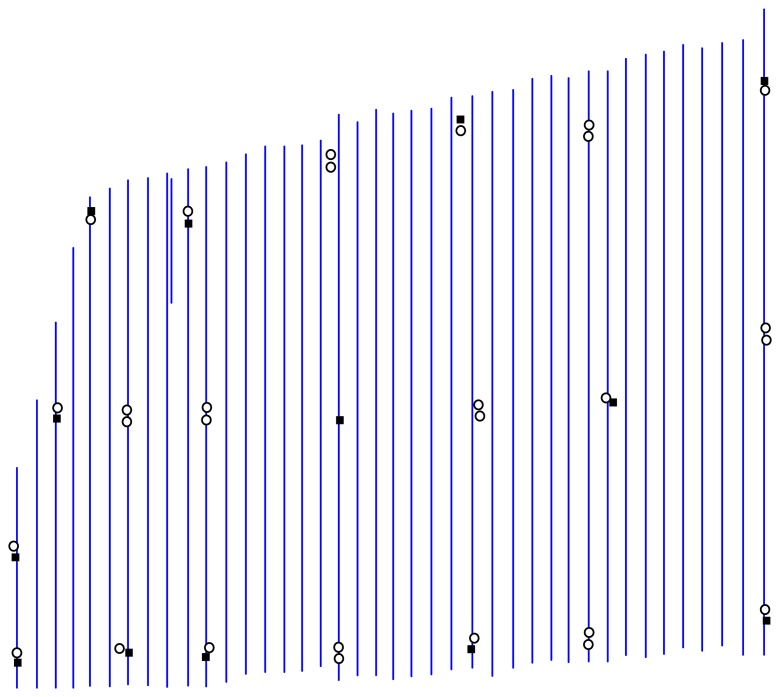
Distribution of flight lines, GCPs and check points of an ADS40 project with 41 strips. ▪ means control point, ○ means check point.

To fully demonstrate the advantages of using the *ω-φ-κ* system against the quaternions, a large project with 41 ADS40 strips that covered more than 7,000 km^2^ was used for experiments. The project was located in Henan Province (China). The 41 strips' images with 0.45 m GSD were acquired by three flight missions. The amount of automatically matched conjugate points by DPGrid was more than 300,000 for this test dataset. There were 41 field measured ground points, in which 13 points were used as GCPs and the other 28 points as check points in the bundle adjustment as shown in Figure 2. The size of absolute orientation files (*.odf), which contained the exterior orientation elements of each scanner line, was about 874 MB. There were totally 4,312 orientation images by using the orientation image model with 8 s intervals. The band width of unknowns was 2,196 after optimization. Consequently, the needed memory to store the coefficient matrix of the normal equation was about 434 MB (4,312×6×2,196×8/1,024/1,024 = 434 MB). Our proposed approach and developed software was capable to deal with the large dataset as a whole project with both systematic error compensation model and orientation image model and both gave stable and similar bundle adjustment results. The result of bundle adjustment with orientation image model was listed in Table 5. The RMSE of height residuals was about 1.0 GSD, and the maximum residual in height was about 2.0 GSD. There was no stereo view hardware available while performing this experiment, so it was not possible to precisely measure the image points under stereo view environment. Although the height accuracy was comparatively lower than those of the other three test fields because the image coordinates of GCPs and check points were not precisely measured, it still qualified for the requirements of topographic mapping at scale 1:5,000 in China.

## 4. Conclusions

For airborne three-line scanner imagery, aerial triangulation is a prerequisite of photogrammetric product generation. Rotation angles were adopted in this paper to express the attitudes of the sensor, and mathematical models of systematic error compensation and orientation image were proposed for aerial triangulation. Using the rotation angles against quaternions has the advantages of eliminating the systematic errors effectively and dealing with large datasets.

The results of three test blocks demonstrate that the RMSE values of the check points are smaller than 1.0 GSD in both the planimetry and the height after bundle adjustment with the systematic error compensation model. However, there are still some systematic residuals for the check points since the systematic errors of the GPS/IMU observations could not be fully compensated. The accuracies of the check points in both the planimetry and the height were substantially improved by bundle adjustment with the orientation image model; the RMSE values in the height direction were refined in particular, from 1.0 GSD to 0.75 GSD. The systematic error of the check point residuals was also compensated, which demonstrates the reasonability and superiority of the orientation image model. Generally, 5 to 10 percent improvements were achieved by the combination of the two models when compared with using the orientation image model only. Moreover, dense conjugate points with very good quality automatically matched by DPGrid software also contribute to improve the accuracy of bundle adjustment.

## Figures and Tables

**Table 1.** Error statistics of bundle adjustment of the Taigu test field (Unit: m).

**Point number**	**Coordinates**	**Systematic error compensation model**			**Orientation image model**			**Systematic error compensation and orientation image model**		

		**RMSE**	**Mean**	**Max**	**RMSE**	**Mean**	**Max**	**RMSE**	**Mean**	**Max**

GCPs 4	X	0.057	0.019	−0.073	0.045	0.013	−0.061	0.042	0.013	−0.058
	Y	0.032	0.002	0.054	0.031	0.005	0.055	0.031	0.005	0.056
	Z	0.050	0.010	−0.104	0.044	−0.012	−0.095	0.045	0.011	−0.091

Check points 101	X	0.045	0.021	0.103	0.043	0.015	0.085	0.041	0.013	0.085
	Y	0.039	0.008	−0.115	0.035	0.006	−0.077	0.035	0.006	−0.072
	Z	0.058	−0.015	−0.158	0.046	0.012	0.115	0.043	0.012	0.104

**Table 2.** Error statistics of bundle adjustment of Pingyao test field (Unit: m).

**Point number**	**Coordinates**	**Systematic error compensation model**			**Orientation image model**			**Systematic error compensation and orientation image model**		

		**RMSE**	**Mean**	**Max**	**RMSE**	**Mean**	**Max**	**RMSE**	**Mean**	**Max**

GCPs 4	X	0.105	0.018	0.206	0.093	0.012	0.233	0.091	0.011	0.206
	Y	0.135	−0.010	0.286	0.114	0.020	0.227	0.110	0.016	0.201
	Z	0.164	0.040	−0.323	0.135	0.025	0.272	0.123	0.021	0.237

Check points 80	X	0.129	0.036	0.328	0.105	−0.015	−0.275	0.100	−0.015	−0.275
	Y	0.148	−0.025	−0.332	0.108	0.018	0.282	0.098	0.018	0.252
	Z	0.185	0.048	0.487	0.148	0.022	0.276	0.143	0.021	0.271

**Table 3.** Error statistics of bundle adjustment of Waldkirch test field (Unit: m).

**Point number**	**Coordinates**	**Systematic error compensation model**			**Orientation image model**			**Systematic error compensation and orientation image model**		

		**RMSE**	**Mean**	**Max**	**RMSE**	**Mean**	**Max**	**RMSE**	**Mean**	**Max**

GCPs 4	X	0.124	0.036	0.241	0.101	−0.012	0.215	0.096	−0.012	0.203
	Y	0.113	0.051	−0.213	0.107	−0.031	−0.204	0.112	−0.022	−0.182
	Z	0.142	0.044	0.223	0.125	0.021	0.215	0.121	0.014	0.202

Check points 26	X	0.087	0.048	0.184	0.087	0.037	0.185	0.085	0.032	0.159
	Y	0.088	0.045	0.216	0.078	0.018	0.217	0.076	0.015	0.210
	Z	0.162	−0.070	−0.279	0.149	−0.021	−0.275	0.145	−0.015	−0.253

**Table 4.** Adjustment results from published reference papers [16,17] (Unit: m).

**Test field**	**Point number**	**RMSE**			**Maximum residual**		

		**X**	**Y**	**Z**	**X**	**Y**	**Z**

taigu	4 GCPs 101 Check points	0.07	0.05	0.06	0.12	0.10	0.13
pingyao	4 GCPs 84 Check points	0.15	0.19	0.19	0.45	0.43	0.45
waldkirch	4 GCPs 26 Check points	0.174	0.174	0.304	N/A	N/A	N/A

**Table 5.** Error statistics of bundle adjustment of Henan province test field (Unit: m).

**Number of points**	**RMSE**			**Mean residual**			**Maximum residual**		

	**X**	**Y**	**Z**	**X**	**Y**	**Z**	**X**	**Y**	**Z**

13 GCPs	0.450	0.520	0.464	0.043	0.034	0.065	−1.126	1.052	−0.857
28 Check points	0.296	0.476	0.408	0.012	0.024	0.106	0.734	1.006	0.924
